# Significance of Chromosome 9p Status in Renal Cell Carcinoma: A Systematic Review and Quality of the Reported Studies

**DOI:** 10.1155/2014/521380

**Published:** 2014-04-30

**Authors:** Ismail El-Mokadem, John Fitzpatrick, Bhavan Rai, J. Cunningham, Norman Pratt, Stewart Fleming, Ghulam Nabi

**Affiliations:** ^1^Academic Section of Urology, Medical Research Institute, Ninewells Hospital, University of Dundee, Dundee DD1 9SY, UK; ^2^Department of Cytogenetics, Ninewells Hospital, University of Dundee, Dundee DD1 9SY, UK; ^3^Department of Pathology, Ninewells Hospital, University of Dundee, Dundee DD1 9SY, UK; ^4^Division of Imaging and Technology, Medical Research Institute, Medical School, University of Dundee, Dundee DD1 9SY, UK

## Abstract

Defining the prognosis of renal cell carcinoma (RCC) using genetic tests is an evolving area. The prognostic significance of 9p status in RCC, although described in the literature, remains underutilised in clinical practice. The study explored the causes of this translational gap. A systematic review on the significance of 9p status in RCC was performed to assess its clinical applicability and impact on clinical decision-making. Medline, Embase, and other electronic searches were made for studies reporting on 9p status in RCC. We collected data on: genetic techniques, pathological parameters, clinical outcomes, and completeness of follow-up assessment. Eleven studies reporting on 1,431 patients using different genetic techniques were included. The most commonly used genetic technique for the assessment of 9p status in RCC was fluorescence in situ hybridization. Combined genomic hybridisation (CGH), microsatellite analysis, karyotyping, and sequencing were other reported techniques. Various thresholds and cut-off values were used for the diagnosis of 9p deletion in different studies. Standardization, interobserver agreement, and consensus on the interpretation of test remained poor. The studies lacked validation and had high risk of bias and poor clinical applicability as assessed by two independent reviewers using a modified quality assessment tool. Further protocol driven studies with standardised methodology including use of appropriate positive and negative controls, assessment of interobserver variations, and evidenced based follow-up protocols are needed to clarify the role of 9p status in predicting oncological outcomes in renal cell cancer.

## 1. Introduction

There are a number of challenges in renal cell carcinoma (RCC) management owed to the lack of biomarkers for early diagnosis and prognosis. Approximately 30% of patients have metastasis at the time of diagnosis [1] and 30% develop metastatic disease on followup after radical surgery for clinically localized disease [1, 2]. Metastatic spread has variable natural history with unpredictable response to targeted therapy. On the other hand, the prognosis of locally advanced nonmetastatic RCCs (pT3N0 M0) exhibits a large variation between patients with 50% cancer specific mortality at 5 years. Furthermore, a significant shift in the stage at diagnosis has been observed in the past two decades with more number of small renal masses (SRMs) (<4 cm) being diagnosed [3]. Current methods such as pathological parameters from biopsies, measuring the lesion growth rate on serial cross-sectional imaging, have been shown to be inaccurate for predicting the true natural history of SRMs [4–6]. A consensus realization is emerging, that there is a need for reliable prognostic indicators, which then can be integrated along with other established parameters into a model for risk stratification as well as guiding clinical decision-making.

Cytogenetic subtyping plays an important role in RCC by characterizing sporadic clear cell RCC (ccRCC) with loss of 3p [7, 8] and papillary RCC (pRCC) with gain of chromosomes 7 and 17 [9, 10]. The integration of cytogenetic testing with the histopathology enhances diagnostic accuracy of renal tumour biopsies [11–13].

The prognostic role of genetic aberrations has been explored in many studies investigating chromosomal copy number aberrations (CNAs) in relation to pathological parameters and clinical outcomes [14–16]. One of the most frequent nonrandom chromosomal CNAs confirmed in ccRCC is 9p deletion [17–20]. The significance of chromosome 9p has been reported in several studies and has been suggested as a marker of RCC aggressiveness [7, 21–28]. Two overlapping studies, from the same institution, suggested that integration of 9p status into prognostic models could improve the predictive accuracy of ccRCC specific survival to 89% [16, 29]. There are, however, a number of factors which remain unclear, such as consensus on the genetic method employed to detect 9p status, its clinical applicability, and cost implications. Thus, there is an urgent need to gain insight into the role of chromosome 9p status and its clinical applicability through a systematic synthesis of the reported literature in order to guide health care decision-makers, patients, and organizational managers involved in the care of RCC.

We aimed to systematically appraise and interpret the reported evidence on the prognostic value of chromosome 9p deletion in RCC by following a set of objectives:

- Evaluate the various genetic techniques employed to assess chromosome 9p status in RCC including risk of bias and concerns for clinical applicability.
- Evaluate the correlation between chromosome 9p status and pathological parameters.
- Evaluate the impact of chromosome 9p deletion on disease free survival (DFS) and cancer specific survival (CSS) in RCC.

## 2. Methods

### 2.1. Search Strategy and Study Eligibility Criteria

We undertook a systematic review of the RCC literature published between 1 January 1990 and the last date of search on 25 September 2013 in the online databases such as Medline, Embase, and PubMed. The terms used for search were ((chromosome 9) OR (fluorescence in situ hybridization) OR (comparative genomic hybridization) OR (cytogenetic) OR (microsatellite) OR (karyotyping) OR (9p loss) OR (9p deletion) OR (loss of heterozygosity) OR (sequencing)) AND “renal cell carcinoma” [MeSH] AND (Humans [Mesh] AND English [lang] AND adult [MeSH]). In addition, reference lists were checked for relevant published studies for inclusion. Studies in English language were included, if they evaluated one or more genetic techniques assessing chromosome 9p status in adult participants (age >18) of any gender with any RCC subtype. For clinical outcome assessment, studies with at least 3 years of followup were included. We arbitrarily chose 3 years to allow an estimation of the discriminative ability of the 9p status between those with poor and good oncological outcomes. We excluded case reports and studies reporting on participants with nonrenal tissue or on urothelial carcinoma. The latest report was included if there was more than one report from the same institution, provided methodology was the same and reviewers felt that there is a possible overlap of study participants reported.

### 2.2. Data Analysis and Management of the Included Studies

The reported data in the included studies, such as sample size, inclusion criteria, patient demographics, genetic technique used for assessing deletion of chromosome 9p, validating techniques, clinicopathological parameters, follow-up period, and survival were analyzed.

### 2.3. Quality Assessment

The quality of the included studies was evaluated by three independent reviewers using the “Strengthening the reporting of observational studies in epidemiology (STROBE) tool” [30]. We modified this tool for the purpose of this review and studies were scored as “high risk of bias,” “low risk of bias,” or “unclear risk of bias” and “high concerns regarding applicability,” “low concerns regarding applicability,” or “unclear concerns regarding applicability” for four key domains: patient selection (domain 1), defining threshold of the test (domain 2), validation of test (domain 3), and flow and followup of cohort (domain 4). The “unclear” category was used when insufficient data were reported to permit judgment. We set a list of characteristics of the most ideal test for assessing chromosome 9p deletion (Table 1). To tailor the STROBE tool for studies about chromosome 9p deletion, we adjusted the original signaling questions of the tool according to this list and formulated extra signaling questions to check applicability.

## 3. Results

**Figure 1:**
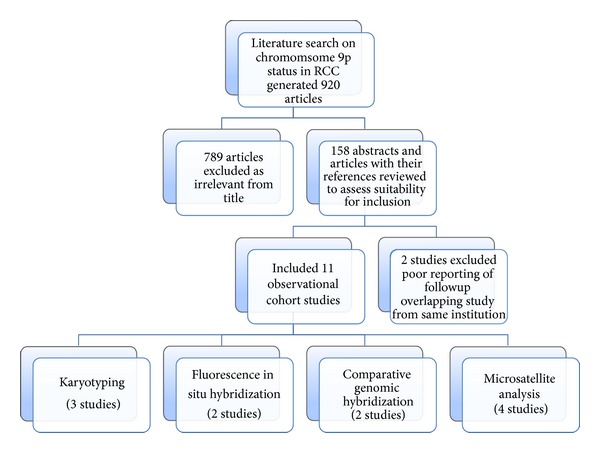
Flow of studies in the review.

Figure 1 shows the process of study selection. We identified 920 abstracts of which 158 were found to be relevant describing one or more genetic technique for assessing 9p deletion in RCC. Of these, 145 studies were excluded, based on the above mentioned criteria. Only 13 studies evaluated 9p status in RCC in relation to pathological parameters and clinical outcomes. We had to exclude one study as it was an earlier report from the same institution with overlapping of cases [16]. We also excluded another study as follow-up data were not clearly stated [41]. Four studies were from the USA, 2 from Germany, 2 from Switzerland, 2 from Italy, and one from China (Table 2).

### 3.1. Characteristics of the Included Studies

Eleven included studies assessed 1431 patients. The quality of reporting in most of the studies for demographic characteristics was poor. Gender and median age of the cohort were reported in five and six studies, respectively (Table 2). Single specialist uro-pathologist assessed the tumours in 9 studies [29, 31–37, 39, 40], and status of report by a specialist pathologist was unclear in the remaining included studies.

Three studies reported on pRCC and 8 studies focused only on ccRCC (Table 2). In 2 studies, the cohort population consisted of patients treated with radical nephrectomy for stage pT3N0 M0 ccRCC [36, 37]. In the remaining 9 studies, there were no limitations on the stages included in the analysis of chromosome 9p status. The data from all the included studies were heterogeneous and correlation between pathological parameters and 9p status was not clearly described in most of the studies.

Different genetic techniques were employed for assessing 9p deletion (Table 3). Briefly, techniques are described below and summarized in Table 3.

#### 3.1.1. Interphase-Fluorescence In Situ Hybridization (I-FISH)

There were 484 tumours assessed for 9p deletion by I-FISH in 4 studies. Brunelli et al. and La Rochelle et al. performed I-FISH on tissue microarrays (TMAs) constructed from FFPE blocks. Each tumour was represented with 3 cores. In addition, at least 1 core of the adjacent normal tissue served as negative control. Each core was 0.6 mm in diameter and 4 to 5 *μ*m thick.

Whereas Schraml et al. performed I-FISH on whole FFPE tissue sections with no negative control [39]. Sanjmyatav et al. used single cell suspension extracted from fresh frozen tumour tissue and 10 from normal kidney tissues as negative control to set threshold for deletion [38].

Studies used dual-fluorescent centromeric probes containing locus specific identifier (LSI) p16 on 9p21 region which is part of CDKN2A gene. Three studies agreed on scoring signals in at least 100 neoplastic interphase nuclei per tumour [32, 38, 39]. Only one study relied on 2 separate investigators for I-FISH interpretation. However, there was no report on the degree of interobserver variation and disagreement was settled by consensus [29]. Only Sanjmyatav et al. mentioned blinding of the I-FISH observer to the final histopathology [38]. There was no consensus standard methodology for defining chromosome 9p deletion in the 4 studies employing I-FISH. Two studies used negative control of normal renal tissue to calculate the threshold for 9p deletion [32, 38] which ranged between 10% and 31%. On the other hand, it was completely arbitrary in the other 2 studies ranging between 40% and 50% [29, 39]. I-FISH was the sole technique employed in 2 studies with no confirmation by subsequent validating technique [29, 32]. I-FISH was used as an adjunct technique in the other 2 studies with good concordance with array CGH results in one study [38].

#### 3.1.2. Comparative Genomic Hybridization (CGH)

CGH was the main technique to assess chromosomal aberrations in 94 patients with ccRCC in 2 studies (Table 3). One study used conventional CGH [36] and the other used array CGH [38]. DNA was extracted from fresh frozen tissue in one study [38]. In the other study, it was extracted from both fresh frozen tissue and FFPE sections [36]. The 2 studies identified frequent similar nonrandom chromosomal CNAs including loss of 3p, 9p, 8p, and 14q and gain of 5q and 7q. Sanjmyatav et al. [38] used I-FISH for validation of array CGH results with strong agreement and correlation with metastasis and CSS.

#### 3.1.3. Microsatellite Analysis

Microsatellite analysis was used as the main technique in 4 studies to assess 284 RCC (ccRCC *n* = 253, pRCC *n* = 31) for chromosome 9p deletion [35, 37, 39, 40]. Two hundred and twenty-eight cases out of 284 cases (80%) were informative for microsatellite analysis with survival data. In all the included studies for this technique, analysis of allelic deletions or instability was performed using a range of 2 to 4 polymorphic microsatellites on chromosome 9p (Table 3). One study assessed loss of heterozygosity (LOH) in other chromosomal regions besides 9p, such as chromosomes 3p, 8p, and 14q [37].

In all the studies, allelic loss was scored if the signal from one allele was >50% reduced in the tumour DNA compared with the controls. The presence of new, shifted alleles or the appearance of new bands was considered as instability.

Li et al., using quantitative PCR, showed significantly reduced expression of PTPRD in tumours exhibiting instability or LOH at D9S168 locus (9p22-23) with good concordance [35]. In the other study, several adjunct techniques were employed but no reporting on concordance [39].

#### 3.1.4. Karyotyping (Cytogenetic Profiling)

There were 634 tumours investigated using karyotyping for 9p deletion in 4 studies. Hundred and fifteen were pRCC [33, 34] and 519 were ccRCC [29, 31]. Two studies were reported from the same institution [29, 34]. In all 4 studies, viable tumour samples were obtained immediately after surgical extirpation and dissected before being dissociated with collagenase II. Cells were washed, cultured, and harvested according to the authors' standard protocol. At least 20 metaphases were analyzed in accordance with the International Standing Committee on Human Cytogenetic Nomenclature by single cytogeneticist.

#### 3.1.5. Sequence Analysis

Schraml et al. [39] assessed CDKN2A sequence alterations in 113 ccRCC as an adjunct technique to microsatellite analysis. They detected 24 bp deletion within exon 1 of CDKN2A in 12% of the tumours which did not correlate with pathological parameters or cancer specific survival. They reported a homozygous G to C trans-version in Exon 3 of CDKN2A in 78.7% of cases, which correlated with higher tumour grade. The authors did not report on concordance with microsatellite analysis or I-FISH.

### 3.2. Detection Rate of Chromosome 9p Deletion in RCC

Table 3 shows the detection rate of chromosome 9/9p loss in all the included studies based on genetic techniques used. 9p loss ranged between 13% and 36.9% in ccRCC and between 9% and 22% in pRCC.

### 3.3. Correlation between Chromosome 9p Status and Pathological Parameters

Six studies assessed the relationship between chromosome 9p loss, grade, and stage of tumour specimens [29, 33–35, 39, 40] (Table 4). For pRCC, two studies showed that 9p loss was significantly associated with higher stage and the more aggressive type II pRCC [33, 34]. On the other hand, 9p loss was significantly associated with higher grade in one study [40].

Whereas, in 2 studies reporting on ccRCC, 9p loss was significantly more common in higher stage tumours [29, 35]. La Rochelle et al. also found correlation between 9p loss and higher Fuhrman grades (G3/G4) [29]. Sanjmyatav and colleagues also showed that loss of the region 9p21.3p24.1 on array CGH and I-FISH was significantly associated with the presence of metastasis [38]. Controversially, Schraml et al. found no association between stage, grade, and LOH at 9p21 [39]. Table 4 summarizes the studies which correlated pathological parameters with 9p status.

### 3.4. Followup

Follow-up period in all the studies was evaluated from the date of surgical extirpation to the last known followup or death. Follow-up data were available for 1,346 out of 1,431 cases (94%). The median followup ranged between 31 and 73 months. Only in 4 studies, the authors followed a standardized protocol for patients' followup [29, 31, 35, 36].

### 3.5. Chromosome 9p Status and Clear Cell RCC Prognosis

In 7 ccRCC studies, 9p deletion was associated with worse outcomes, including being an independent prognostic factor in 3 studies on multivariate analysis [29, 32, 35]. On the other hand, Antonelli et al. and Presti et al. demonstrated that 9p deletion had no impact on ccRCC prognosis (Table 2). Two studies showed that localized ccRCC with 9p deletion carried a significantly higher risk of recurrence and cancer related deaths compared to nondeleted tumours [29, 35]. The 5-year DFS for 9p deleted tumours ranged between 26% and 50% compared to 71% and 98% in tumours without 9p deletion. Also, 5-year CSS ranged between 28% and 67% for 9p deleted compared to 87%–98% for nondeleted 9p tumours (Table 2). On multivariate analysis models, 9p loss was an independent prognostic factor for both DFS and CSS in localized ccRCC in one study (*P* = 0.15) [29, 35] and D9SS168 alterations (LOH or instability) on 9p was an independent prognostic factor for CSS in another study (*P* = 0.009) [35]. Two studies concluded that 9p loss in pT3N0 M0 ccRCC tumours was associated with worse DFS [36] and CSS [39] only on univariate analysis. Controversially, Presti et al., in a larger cohort of patients with pT3N0 M0 ccRCC tumours, noticed a trend towards worse DFS in patients with 9p deleted tumours but this did not reach statistical significance [37]. Only in one study with the largest number of cases, a subset analysis of 207 patients with SRMs (<4 cm) was undertaken. 9p deleted tumours were associated significantly with lymph node and distant metastasis (*P* = 0.03). The 5-year CSS and DFS were 56% and 68%, respectively, for 9p deleted tumours compared to 90% and 97% for the nondeleted ones (*P* = 0.01). 9p loss had an independent effect on DFS on multivariate analysis model including T-stage, grade, and size (Hazard ratio 6.65; *P* = 0.021) [29]. More recently, Sanjmyatav et al. [38] assessed 53 ccRCCs (31 of which were metastatic) to identify recurrent chromosomal aberrations associated with metastatic disease. Loss of 9p21.3p24.1 was the most prominent of these aberrations with the highest odds ratio for metastatic risk and was linked to shorter CSS only on univariate analysis. However, La Rochelle and colleagues concluded that 9p status had no effect on CSS in cases with metastatic disease at diagnosis (HR, 1.02; *P* = 0.9) [29].

### 3.6. Chromosome 9p Status and Papillary RCC Prognosis

In pRCC, 5-year overall survival was 40% for patients with LOH of D9S171 on 9p compared to 81% for patients without deletion (*P* = 0.008) [40]. This deletion was an independent predictor of prognosis on multivariate analysis including for stage and grade. This study was limited by small number of cases and lack of differentiation between the 2 subtypes of pRCC. Klatte et al. and Gunawan et al., in an attempt to characterize both subtypes of pRCC cytogenetically, detected 9p loss in few cases who seemed to have higher risk of recurrence and cancer specific death, but only on univariate analysis.

### 3.7. Assessment of Bias and Applicability

The STROBE assessment (Table 5) demonstrates that all the included studies had a high risk of bias, or high concerns regarding applicability in all four domains. In general, most studies had a high risk of bias in domain 1 of patient selection and had high concerns regarding applicability, in domains 4A and 4B, the domains concerning the sample size and tissue samples used for the analysis.

Only two studies chose consecutive cases as their sample [29, 39]. For the rest of the studies, method of recruitment was not clearly mentioned.

All the studies determined clearly the cut-off threshold for the main technique employed before reporting the results. However, there was a high concern regarding the various arbitrary methods by which the cut-off threshold for 9p deletion was decided in studies especially in those studies using I-FISH as the main technique [29, 32].

Lack of validation was another limitation which could have introduced bias especially when the main technique tended to be observer dependent. Validation for 9p status took place only in 2 studies [35, 38]. High risk of bias could have also been introduced in case selection and clinical followup due to missing data and lack of standard follow-up protocol in some studies. The noninformative cases on microsatellite analysis had to be excluded from survival analysis. Also the validating technique was not employed on all cases. In terms of applicability, there was high concern regarding cohort selection, technique, interpretation of results, and threshold decision in all the included studies as summarized in Table 5.

## 4. Discussion

Our systematic appraisal of the literature summarizes the evidence for 9p status and its clinical applicability in RCC. The ideal study on chromosome 9p status in RCC has yet to be performed to answer key clinical questions. All the studies included in this review were retrospective observational in nature with small cohorts of patients, except in one study, where numbers were sufficient to allow subset analysis [29]. Moreover, there was a lack of reporting on measures to avoid bias such as using consecutive cases, number of specialized pathologists to characterize tumours, blinding the assessor of 9p status to pathological parameters and clinical outcome. It was not possible to assess collectively the correlation between 9p status, pathological parameters, and survival due to lack of reporting on demographic characteristics, the heterogeneity of the reported data including pathological parameters, various genetic techniques, different tissue used, variations in cut-off thresholds, and follow-up protocols.

Six studies reported a correlation between 9p deletion and RCC aggressiveness (stage and grade) (Table 4). Chromosome 9p loss was associated with worse survival in 9 studies. However, when included in multivariate analysis, it had only an independent effect in three studies reporting on ccRCC and one study on pRCC (Table 2). Also, integration of 9p status in prognostic models for ccRCC improved the predictive accuracy of CSS up to 89% in 2 studies from the same institution [16, 29].

Several techniques were used to assess 9p deletion; each had its own advantages and limitations. I-FISH can be applied on either fresh or FFPE tissue. It assessed chromosomal CNAs at a single cell level. However, I-FISH scoring was more likely to be influenced by fading of signals, overlapping of cells, and angle of tissue slicing from paraffin blocks, which could result in interobserver variability. In addition, the lack of standardization of scoring with different cut-off thresholds used to determine 9p loss represents a major challenge to the applicability of I-FISH in a clinical setting [42].

Microsatellites are useful in detecting LOH of a locus or gene; however, more microsatellites are required to reliably detect chromosomal CNAs. Also, one out of five cases tested for 9p deletion by microsatellites in this review was noninformative and had to be excluded from analysis.

In contrast to I-FISH and microsatellites, Karyotyping and CGH assess the whole genome for chromosomal abnormalities which could be implicated in tumour progression. However, Karyotyping relies on cell culture from fresh tumour tissue, with potential culture failure rate ranging between 10 and 12% [16, 31]. It can underestimate 9p loss due to chromosomal condensation of mitoses, lack of precision to detect microdeletions, and frequent complex rearrangements [43]. CGH is more sensitive for chromosomal CNAs due to its high resolution. However, it can miss translocations. Also, due to bulk extraction of the DNA, genomic heterogeneity information can be diluted or masked [44].

Some genes on chromosome 9p have been suggested to play a role in ccRCC progression. However, none of them has been confirmed to be the rate limiting gene. The low level of expression of carbonic anhydrase IX (CAIX) gene located on locus 9p13 by immunohistochemistry was shown to be associated with worse prognosis [14, 45, 46]. Also low levels of p16INK4a protein expression, which is encoded by cyclin-dependent kinase inhibitor 2A (CDKN2A) gene located on 9p21, had an effect on prognosis [47, 48]. Li et al. reported that D9S168 microsatellite alterations, located at 9p23-24 gene region, which encodes for protein tyrosine phosphate receptor delta (PTPRD), could contribute to worse prognosis [35]. The process of RCC progression is complex involving various genetic and epigenetic events. Results from genome sequence analysis helped to closely study and identify new mutations within genes implicated in RCC progression such as PBRM1, SETD2, and BAP1 [19, 49, 50].

### 4.1. Implications for Clinical Practice

The review showed that the reported evidence was not strong enough for the translation of 9p status genetic testing into clinical practice. The reported literature showed a high concern for bias and applicability. We estimated that it will take a few years until chromosome 9p status can be integrated as a part of routine clinical practice for every patient with RCC in the public, social insurance health-care system. The technique has to be reliable, cost-effective, timely, and of equitable manner before being adopted widely.

### 4.2. Implications for Research

A number of recommendations could be made based on our study.

- Protocols for genetic techniques needs to be standardized and thresholds for deletion agreed upon. Tissue sampling needs to be assessed with tumour genomic heterogeneity kept in mind to reduce sampling bias. Also interobserver agreement should be reported.
- The results of new and emerging molecular genetic techniques need to be validated using a complementary technique including appropriate use of negative and positive controls.
- Further well-designed prospective studies are required to confirm the role of 9p deletion in RCC progression and prognosis.
- Further studies are required to identify the rate limiting genetic aberration on chromosome 9p implicated in clinical progression of the disease in the context of genetic and epigenetic factors.

## 5. Conclusion

The evidence for chromosome 9p status in RCC is emerging mainly focusing on its value in predicting clinical outcomes; however, a number of concerns regarding methodology of research, quality of reporting, and its applicability in clinical practice exist. Future research is needed to address these issues.

## Figures and Tables

**Table 1:** STROBE checklist and signaling questionnaire used in the chromosome 9p studies in renal cell carcinoma modified by members of the Tayside Urological Cancers Network (TUCAN).

Domain	(1) Patient selection	(2) Definition of threshold of the test	(3) Use of validation test	(4) Flow and clinical followup
Signaling questions (yes/no/unclear)	*Was a consecutive or random sample of patients enrolled?* Yes, if this information was described in the article	*If a threshold was used, was it prespecified?* Yes, if cut-off values were explained before data analysis	*Were the results confirmed by a second validation test? * Yes, if a second test was applied to confirm the results	*Was there an appropriate clinical outcome/pathological outcome used in the study* Not assessed
	*Was a case-control design used?* Yes, if a case-control design was used		*Were the validation results interpreted without knowledge of the results of the original test?* Yes, if genetic technique results were interpreted without knowledge of the second validation test results	*Did all patients receive genetic test? (partial verification avoided?)* Yes, if all patients received a genetic test
	*Did the study avoid inappropriate exclusions?* Yes, if no sample was excluded based on clinical stage or grade of renal tumor			*Did all patients receive the same validation test? *(*differential verification avoided?*) Yes, if all patients the same validation test
				*Were all patients included in the analysis?* Yes, if all patients were included and no patients were reported as unclassified

Risk of bias (high/low/unclear)	Conclusion: could the selection of patients has introduced bias?	Conclusion: could the conduct or interpretation of the genetic technique has introduced bias?	Conclusion: could the validation test, its conduct, or its interpretation have introduced bias?	Conclusion: could the patient flow/followup have introduced bias?

Concerns regarding applicability (high/low/unclear)	(1) *Are there concerns that the included patients do not match the review question?* Low, if results confirm that patients were included as defined by clinical stage of the disease	(2A) *Are there concerns that the genetic technique, its conduct, or interpretation differ from the review question?* Low, if the genetic technique was performed to answer clinically relevant outcomes as defined in the review (2B) Was one threshold used, or in case the results can fall in between two thresholds (and is inconclusive). Yes, if one of these situations was true.	(3) *Are there concerns that the target condition as defined by the genetic technique does not match the review question? * Low, if genetic technique of chromosomal 9p status did match stage and grade of the disease	**Additional question 1** (4A) *Are there concerns about the sample size of the cohort?* Low, if the cohort was selected with proper statistical power to answer clinically relevant outcome **Additional question 2** (4B) *Are there concerns about the tissue samples used for genetic technique?* Low, if the tissue samples were obtained before the more invasive and potentially harmful procedure such as radical nephrectomy

**Table 2:** Summary of the included studies.

Study/country	RCC subtype	Total number of cases	Median age (years)	Cases with followup assessed for 9p status	cN+M+ *n*= (%)	Median follow-up months	Follow-up protocol described	Primary outcome analysed	5-year survival		*P* value
									9p deleted	9p retained	
(1) Antonelli et al., 2010 [31] ITA	ccRCC	131	62.9 (mean)	131	19 (14.5%)	73	Yes	CSS	60%	78%	0.312

(2) Brunelli et al.,2008 [32] ITA	ccRCC	73	65	73	7 (10%)	45	No	CSS	43%	88%	<0.001*

(3) Gunawan et al., 2003 [33] GER	pRCC	50	62	38	9 (24%)	41	No	DFS	NS	NS	0.00003

(4) Klatte et al., 2009 [34] USA	pRCC	65	61	57	NS	39	No	CSS	0%	80%	0.009*

(5) La Rochelle et al., 2010 [29] USA	ccRCC	703	NS	703	260 (37%)	Mean 40	Yes	DFS CSS (only N0M0)	49% 67%	77% 87%	<0.001* <0.014*

(6) Li et al., 2011 [35] CHINA	ccRCC	93	55.5	78	0%	31.7	Yes	DFS (LOH) CSS (LOH)	26% 28%	98% 98%	0.001 0.001*

(7) Moch et al. 1996 [36] SWI	ccRCC	41 (pT3)	NS	37	0%	39	Yes	DFS	31%	70%	0.04

(8) Presti et al., 2002 [37] USA	ccRCC	72 (pT3)	NS	67	0%	41	No	DFS	50%	71%	0.14

(9) Sanjmyatav et al., 2011 [38] GER	ccRCC	53	61	53	31 (58%)	47	No	CSS	0%	75%	0.00001

(10) Schaml et al., 2001 [39] SWI	ccRCC	113	NS	88	12 (14%)	48	No	CSS (only pT3) (*n* = 37)	LOH 0%	58%	0.01

(11) Schraml et al., 2000 [40] SWI	pRCC	37	NS	21	NS	54	No	OS	40%	81%	0.008*

ccRCC: clear cell renal cell carcinoma; pRCC: papillary renal cell carcinoma; NS: not stated clearly; CSS: cancer specific survival; DFS: disease free survival; OS: overall survival; LOH: loss of heterozygosity; *Statistically significant on multivariate analysis.

**Table 3:** Prevalence of chromosome 9/9p deletion according to technique and candidate locus or gene studied.

Study	Genetic technique	Threshold for deletion	Candidate locus/gene	Number of 9p deleted cases (%)
(1) Antonelli et al., 2010 [31]	Karyotyping	20 metaphases*	Chromosome 9/9p	19/131 (14.5%)

(2) Brunelli et al., 2008 [32]	FISH	Mean + 4SD the percentage of abnormal nuclei in normal tissue → 30% for monosomy and 31% for LOH	LSI p16 (INK4A,B, ARF)	13/73 (18%)

(3) Gunawan et al., 2003 [33]	Karyotyping	20 metaphases*	Chromosome 9/9p	8/50 (16%)

(4) Klatte et al., 2009 [34]	Karyotyping	20 metaphases*	Chromosome 9/9p	6/65 (9%)

(5) La Rochelle et al., 2010 [29]	FISH	If majority of nuclei were abnormal	LSI p16 (INK4A,B, and ARF)	44/316 (13.9%)
	Karyotyping	20 metaphases*	Chromosome 9/9p	53/388 (13.6%)

(6) Li et al., 2011 [35]	Microsatellite analysis	The signal from one allele was >50% reduced in tumour DNA compared with normal control The appearance of new bands defined as instability (MSI)	D9S168 (9p23–9p24.2) D9S171 (9p21) D9S157 (9p22) D9S1749 (9p21)	34/92 (36.9%) 4/29 (13.7%) 3/23 (13%) 1/27 (3.7%)

(7) Moch et al., 1996 [36]	CGH	Mean green : red ratio ±1 SD	Chromosome 9/9p loss	10/41 (24%)

(8) Presti et al., 2002 [37]	Microsatellite analysis	The signal from one allele was >50% reduced in tumour DNA compared with normal control	D9S925 (9p22) D9S921 (9p22)	21/67 (31%)

(9) Sanjmyatav et al., 2011 [38]	Array CGH	Mean green : red ratio ±1.5 SD	9p21.3 p24.1	11/53 (21%)
	FISH	Mean − 2SD the percentage of normal diploid cells per 10 normal tissues → 10%	LSI p16 (9p21)	Described as 89% sensitivity

(10) Schraml et al., 2000 [40]	Microsatellite analysis	The signal from one allele was >50% reduced in tumour DNA compared with normal control	D9S970 (9p12-9p13)	2/25 (8%)
			D9S171 (9p13)	6/29 (21%)
			D9S1748 (9p21)	2/32 (6%)
			D9S156 (9p21)	1/25 (4%)
			Allelic deletion in 9p with at least one microsatellite →	8/37 (22%)

(11) Schraml et al., 2001 [39]	Microsatellite analysis	The signal from one allele was >50% reduced in tumour DNA compared with normal control	D9S970 (9p12-9p13)	5/68 (7%)
			D9S171 (9p13)	12/59 (20%)
			D9S1748 (9p21)	7/73 (10%)
			D9S156 (9p21)	8/68 (9%)
			Allelic deletion in 9p with at least one microsatellite →	21/88 (24%)
	FISH	40% monosomy and 50% LOH	CDKN2A	6/54 (11%)
	Sequence analysis		CDKN2A → 24 bp deletion within exon 1	13/113 (12%)

LSI: locus specific identifier.

*Analysis in accordance with the International Standing Committee on Human Cytogenetic Nomenclature.

**Table 4:** Correlation between pathological parameters and chromosome 9p status.

Study	Pathological parameters	Chromosome 9/9p status (*n*=) or (%)		Fisher's exact test
		9p loss	9p retained	*P*=
(1) Schraml et al., 2001 [39]	pT1/2	12	29	0.27
	pT3/T4	9	38	
	N0M0	19	57	0.53
	N+M+	2	10	

(2) La Rochelle et al., 2010 [29]	pT1/2	38	349	<0.01
	pT3/T4	59	257	
	N0M0	43	400	<0.01
	N+M+	54	206	

(3) Schraml et al., 2000 [40]	pT1/2	4	9	0.054
	pT3	2	7	

(4) Li et al., 2011 [35]	pT1/2	12	40	0.007
	pT3/4	14	12	
	N0	23	46	1
	N+	3	6	

(5) Gunawan et al., 2003 [33]	G1/2	6	33	Not assessed
	G3/4	2	4
	pT1/2	3	31
	pT3/4	4	6	0.004
	N0M0	3	31	
	N+M+	5	5	0.04

(6) Klatte et al., 2009 [16]	pT1/2	Not stated clearly		0.001
	pT3/4			
	N+	50%	14%	0.027
	M+	67%	14%	0.002

**Table 5:** Assessment of bias and applicability of the included observational cohort studies.

Study	Risk of bias				Applicability concerns			
	Patients selection	Definition of threshold	Validation test	Flow and clinical followup	Cohort selection	Technique interpretation and threshold	Target condition	Sample size and tissue samples used
Antonelli et al., 2010 [31]	High	Low	High	Low	Low	Low	Low	High
Brunelli et al., 2008 [32]	High	Low	High	Low	Low	Low	Low	High
Gunawan et al., 2003 [33]	High	Low	High	High	Low	Low	Low	High
Klatte et al., 2009 [34]	High	Low	High	High	Low	Low	Low	High
La Rochelle et al., 2010 [29]	Low	Low	High	Low	Low	Low	Low	High
Li et al., 2011 [35]	High	Low	Low	High	Low	Low	Low	High
Moch et al., 1996 [36]	High	Low	High	High	Low	Low	low	High
Presti et al., 2002 [37]	High	Low	High	Low	Low	Low	Low	High
Sanjmyatav et al., 2011 [38]	High	Low	Low	Low	Low	Low	Low	High
Schraml et al., 2000 [40]	Low	Low	High	High	Low	Low	Low	High
Schraml et al., 2001 [39]	Low	Low	High	High	Low	Low	Low	High
